# Nivolumab-Induced Immune Mediated Colitis Localized to the Distal Colon: Seven Years Into Therapy

**DOI:** 10.7759/cureus.72373

**Published:** 2024-10-25

**Authors:** Christian J Nassif, Imad I Nassif, Mouna Todorov

**Affiliations:** 1 Medicine, Kansas City University of Medicine and Biosciences, Kansas City, USA; 2 Gastroenterology, University of Kansas School of Medicine, Wichita, USA; 3 Pathology, Heartland Pathology Laboratories, Wichita, USA

**Keywords:** distal colon, immune-checkpoint inhibitors, immune-mediated colitis, late onset, nivolumab, ulcerative colitis (uc)

## Abstract

Nivolumab targets programmed cell death protein 1 (PD-1) and is used in cancer treatment by increasing the immune response. It has rarely been associated with immune-mediated colitis (IMC). We present a 66-year-old man with metastatic lung adenocarcinoma, treated with nivolumab for seven years, who developed tenesmus and mucoid bloody diarrhea. Colonoscopy revealed endoscopic and histopathological ulcerative colitis (UC)-like changes, limited to the distal 25cm of the colon. Although nivolumab-induced IMC resembling UC is a known adverse effect, there are very few reports of nivolumab-induced IMC localized to the distal colon and occurring seven years after treatment initiation.

## Introduction

Nivolumab is a type of monoclonal IgG4 antibody used to treat many types of cancers, including non-small cell lung cancer [1,2]. It binds programmed cell death protein 1 (PD-1) expressed in T-cells, stimulating the body's T-cell response [2]. As a result, nivolumab is known to rarely cause immune-related adverse effects, including immune-mediated colitis (IMC) [3-5]. IMC and ulcerative colitis (UC) findings are similar and may be indistinguishable in histopathology and colonoscopy [4]. Distal nivolumab-induced IMC is a rare occurrence, with few cases reported. Most cases of nivolumab-induced IMC present as a pancolitis or left-sided colitis with onset occurring several days to two years after nivolumab treatment initiation [4,6,7].

## Case presentation

**Figure 1:**
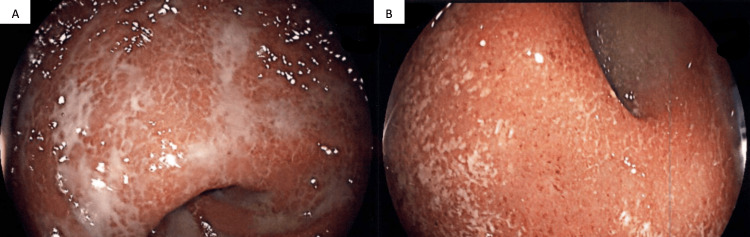
Endoscopic findings in the distal 25 cm of the colon: continuous mucosal edema, erythema, friability, loss of vascularity, shallow reticular ulcerations (A) and innumerable punctated ulcerations (B).

**Figure 2:**
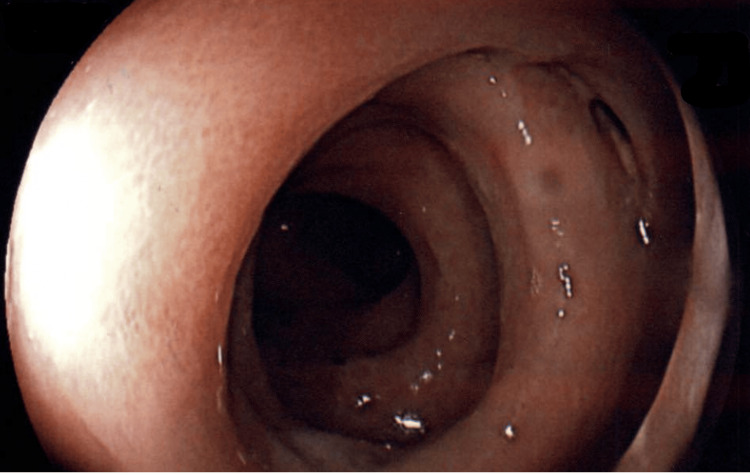
Endoscopically normal colonic mucosa proximal to 25 cm.

**Figure 3:**
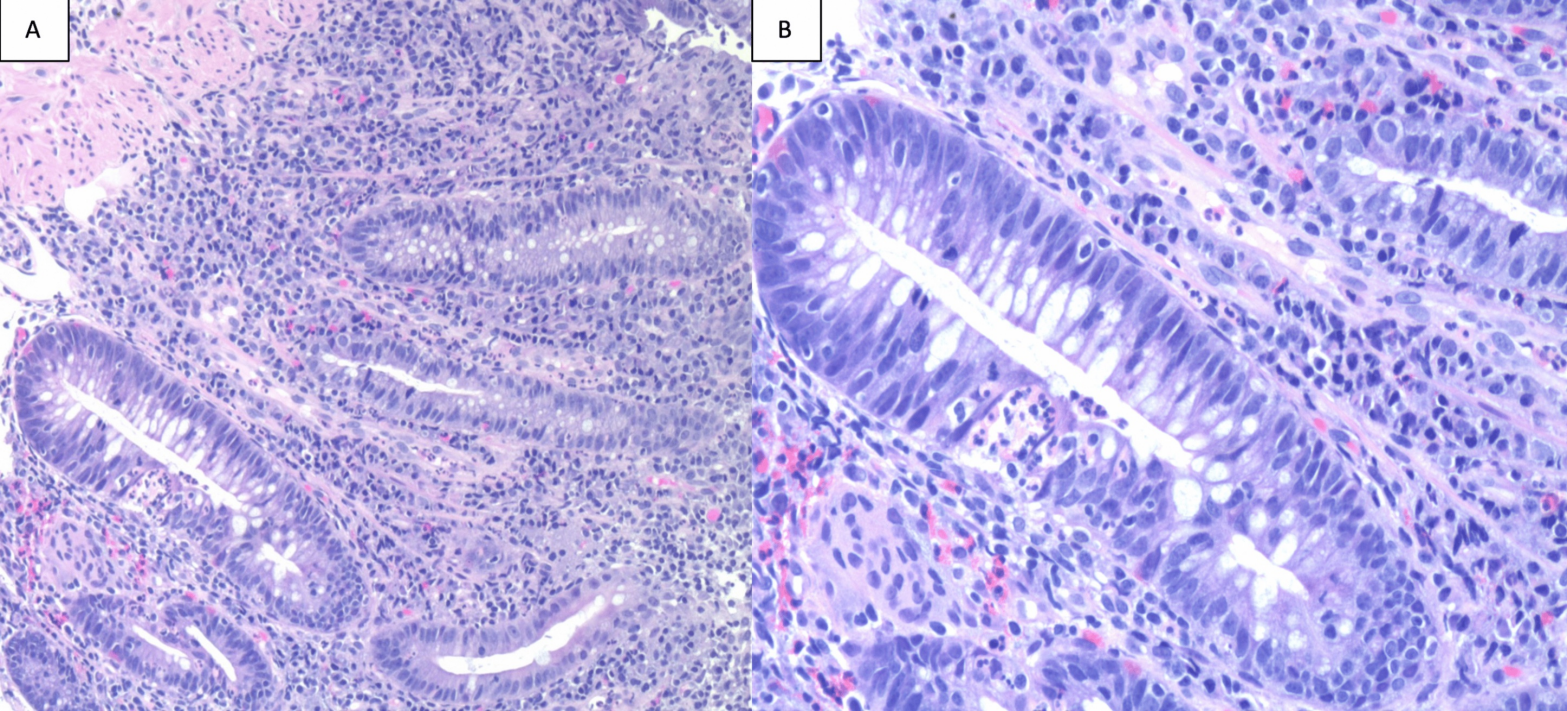
Biopsies of the distal 25 cm of colon, histopathologic findings. Hematoxylin and Eosin staining, 100x (A) and 400x (B). Changes of cryptitis, neutrophilic crypt microabscesses, crypt atrophy, and focal architectural distortion. The lamina propria shows an overall increase in lympho-plasmacytes. Findings are consistent with chronic active colitis.

A 66-year-old man was diagnosed with poorly differentiated metastatic adenocarcinoma of the lung nine years prior to presentation to the digestive diseases clinic. The initial lesion on chest computerized tomography (CT) was a 10cm mass in the right upper lobe of the lung with mediastinal invasion, pleural invasion, pre-tracheal lymph node involvement, and ipsilateral hilar lymph node involvement. Ipsilateral malignant pleural effusion was present and confirmed by thoracentesis. Positron emission tomography (PET) findings correlated with CT findings. After first-line therapy and consolidation treatment, repeat CT and PET scans showed disease progression and contralateral nodules. Second-line therapy was initiated, but CT and PET scan follow-up showed further progression. Nivolumab therapy was initiated. The response to nivolumab was excellent, with marked improvement on CT and PET scans within three months, which continued. Findings on follow-up CT scans and PET scans in the last five years have been negative for residual illness. In the last three years, he had intermittent and few episodes of increased frequency of bowel movements, tenesmus, and mucoid stools, at times awakening him at night. Thirteen months before his presentation, he started losing weight. He had not reported his symptoms until then, as they had not bothered him. Three months before presentation, he noted significantly more frequent and persistent symptoms, accompanied by a total of thirteen pounds of weight loss. Eventually, his diarrhea became bloody, with hematochezia and fresh blood in the stool. A colonoscopy was performed and revealed continuous mucosal edema, erythema, friability, loss of vascularity, shallow reticular ulcerations, and innumerable punctated ulcerations in the distal 25cm of the colon (Figure 1). The terminal ileum and the colon, proximal to 25cm, were normal on endoscopic examination (Figure 2) and histopathological sampling. Biopsies of the distal 25cm of the colon revealed changes of chronic active colitis with cryptitis, neutrophilic crypt microabscesses, crypt atrophy, and focal architectural distortion. The lamina propria showed an overall increase in lympho-plasmacytes (Figure 3). Treatment with oral corticosteroids was initiated with Prednisone at 40mg per day. A very good response and resolution of symptoms occurred within several weeks of therapy. Maintenance oral mesalamine treatment was started and continued. Nivolumab was held.

## Discussion

Nivolumab is an immunotherapeutic agent used for the treatment of non-small cell cancer [1,2] and also many other malignant neoplasms. It is an IgG4 monoclonal antibody that targets PD-1. The purpose of its usage in cancer treatment is to increase the patient's immune response [8]. Nivolumab binds PD-1, which is expressed in T-cells. This stimulates the body's T-cell response [2], providing an anti-neoplastic effect. Nivolumab has been associated with many immune-mediated adverse effects, including pneumonitis, colitis, endocrine disorders, hepatitis, hepatotoxicity, nephritis, renal dysfunction, dermatological disorders, and myocarditis [9]. IMC induced by nivolumab is rare and overall has an incidence rate of 0.7%-2.9% [6,9]. After the first infusion of nivolumab, the onset of IMC occurs after several days to two years, with a median onset of seven weeks [4,6,7,9]. In the case we present, the patient had an excellent response to nivolumab after failing several other treatment protocols for his metastatic adenocarcinoma of the lung. His diagnosis was made rather uncharacteristically nearly seven years after initiation of nivolumab therapy.

Additionally, some reports describe nivolumab-induced IMC as presenting with endoscopic findings of left-sided colitis in 31%-43%, pancolitis in 23%-40%, or ileitis in 11%-14% [4,7]. Nivolumab-induced IMC localized only to the distal 25 cm of the colon, such as in the case we present, is extremely rare, with our own literature review revealing no previously reported cases. PD-1 blockade aims at late T-cell proliferation, therefore causing a more localized immune reaction [7]. It is, therefore, conceivable that nivolumab-induced IMC may be localized only to the distal colon, in this case, the rectum and the recto-sigmoid junction.

The very rare late and distal presentation may be a reason to wonder if this was truly nivolumab-induced IMC or an altogether new onset of idiopathic UC. Reports indicate that idiopathic UC is exacerbated by nivolumab due to its mechanism of action [10,11]. Therefore, a localized, relatively mild, distal idiopathic UC would be unexpected in the case presented. A more widespread and severe colitis would be more likely. The patient also had no history of idiopathic UC before nivolumab treatment. Additionally, clinical improvement started within a few days of nivolumab discontinuation, more rapidly than the improvement expected with oral corticosteroids in UC. Therefore, this case is likely a very rare nivolumab-induced IMC localized to the distal colon seven years into therapy.

The differentiation between IMC and idiopathic UC is exceedingly difficult based on endoscopic and histopathological findings. Both entities may present endoscopic features of continuous mucosal edema, erythema, friability, loss of vascularity, shallow reticular ulcerations, and innumerable punctated ulcerations. Histopathologically, features of chronic active colitis, including cryptitis, neutrophilic crypt microabscesses, crypt atrophy, architectural distortion, plasmacytosis, and neutrophilic infiltrates, may be present in both illnesses [3].

Treatment with corticosteroids and Mesalamine can be effective in both cases, and in more severe presentations, using anti-TNF antibodies may be necessary. In nivolumab-induced IMC, discontinuation of therapy may be necessary in severe cases [7]. In our case, nivolumab was held despite the presentation being consistent with only a grade II colitis, which would not necessarily require withholding nivolumab. This decision was due to the excellent response of the lung's metastatic adenocarcinoma, the complete resolution of findings on CT and PET scans, and the evidence of disease freedom in the last five years.

## Conclusions

Although cases of nivolumab-induced IMC are well documented, cases involving only the distal colon are far more uncommon. Additionally, the occurrence of nivolumab-induced IMC is reported in the literature as occurring up to two years into therapy. This makes the onset of IMC after seven years, as in this case, an exceedingly unusual occurrence. This case underscores the importance of physician awareness of the possible but rare, late, distal, and mild presentation of nivolumab-induced IMC. This makes the similarity in features and presentation between idiopathic UC and nivolumab-induced IMC even greater.
